# FDG-PET/CT for the Management of Post-Chemotherapy Residual Mass in Hodgkin lymphoma

**DOI:** 10.3390/cancers13163952

**Published:** 2021-08-05

**Authors:** Andrea Gallamini, Michał Kurlapski, Jan Maciej Zaucha

**Affiliations:** 1 Research and Clinical Innovation Department, Antoine Lacassagne Cancer Centre, 06189 Nice, France; 2 Haematology and Bone Marrow Transplantation Department, Medical University of Gdansk, 80-214 Gdansk, Poland; kurlapski.michal@gmail.com (M.K.); jan.zaucha@gumed.edu.pl (J.M.Z.)

**Keywords:** Hodgkin lymphoma, residual mass, PET

## Abstract

**Simple Summary:**

In the present review the authors report the predictive value of FDG/PET-CT (PET) on treatment outcome of Hodgkin lymphoma patients showing a post-chemotherapy residual mass, based on the published reports of PET-guided consolidation radiotherapy after different-intensity chemotherapy regimens such as ABVD or BEACOPP_escalated_. A special focus will be dedicated to the role of PET for assessing patients with a residual mass during and after immunotherapy with immune checkpoint inhibitors. Finally, the interpretation criteria of PET will be also reviewed, and the role of alternative imaging techniques discussed.

**Abstract:**

In the present review, the authors report the published evidence on the use of functional imaging with FDG-PET/CT in assessing the final response to treatment in Hodgkin lymphoma. Despite a very high overall Negative Predictive Value of post-chemotherapy PET on treatment outcome ranging from 94% to 86%, according to different treatment intensity, the Positive Predicting Value proved much lower (40–25%). In the present review the Authors discuss the role of PET to guide consolidation RT over a RM after different chemotherapy regimens, both in early and in advanced-stage disease. A particular emphasis is dedicated to the peculiar issue of the qualitative versus semi-quantitative methods for End-of Therapy PET scan interpretation. A short hint will be given on the role of FDG-PET to assess the treatment outcome after immune checkpoint inhibitors.

## 1. Introduction

In medical oncology, the treatment response has been traditionally assessed with standard radiological tools such as Contrast-enhanced Computed Tomography (CeCT) to detect the therapy-induced tumor shrinkage, and the criteria for unidimensional Residual Mass (RM) assessment have been categorized in the so-called Response Evaluation Criteria in Solid Tumor (RECIST) criteria [1]. Later, thanks to the systematic use of functional imaging with ^67^Ga^−^Gallium scintigraphy and, most of all, of [^18^F]-Fluorodeoxyglucose Positron Emission Tomography (FDG-PET), it became clear that the dimensional criteria for tumor response assessment, based on changes in tumor size, were imperfect. In several cancers and, most of all, in lymphoma, the chemotherapy-induced tumor shrinkage takes time, and a RM mass can take years after treatment to disappear [2]. In the original observation of Radford et al., a chest radiograph was regularly performed during the clinical follow-up of 110 patients affected by Hodgkin lymphoma (HL) presenting at baseline with a classical mediastinal bulky lesion, treated with chemotherapy and/or radiotherapy [3]. Up to 59% of the residual radiological abnormalities underwent further shrinkage after the first year of follow-up and 45% of them reverted to normal. As a matter of fact, despite the very good response to therapy, treatment of HL can result in a CeCT-detected RM in up to 80% of patients, most frequently in the anterior mediastinum, without any clinical evidence of disease relapse or progression [3,4]. This challenge has been met in the millennium turnaround by functional imaging, notably by PET, in which FDG uptake is sustained by viable neoplastic tissue, independent from changes in tumor size [5].

Fused functional imaging with FDG-PET and CeCT in a single whole-body scanning session (PET/CT) proved superior to CeCT to assess the response to treatment in this clinical context [6,7].

This modern approach to assess the effectiveness of treatment is based on an advanced imaging method, aimed at tracing the glycolytic activity of the cells, that proved able to pick up even minimal residual viable neoplastic tissue at the end of therapy. The peculiar ability of the glucose analog ^18^F-Fluordo-deoxy-d-glucose (FDG) to trace the neoplastic tissue relies on the elevated glycolytic metabolism of the tumor, which proved to be a hundred times higher than the normal tissues, accounting for the well-known “Warburg effect” activity described by the German physiologist Warburg [8]. On the other hand, the HL tissue shows a completely different neoplastic architecture compared to other neoplasms, characterized by a few, scattered neoplastic cells, the Reed-Sternberg and Hodgkin (HRS) cells, embedded in a largely predominant non-neoplastic micro-environment (ME) tissue of “accessory” cells, (ME cells) such as macrophages, T- and B-lymphocytes, eosinophils, plasma cells, and endothelial cells, which are responsible for the immortalization of HRS cells [9]. ME cells are also characterized by a very high glycolytic activity: in vitro cultures of HRS showed that the glycolytic enzymes accounted for more than 40% of the proteins recovered in the surnatant [10]. This characteristic microstructural architecture underpins the superior performance of functional imaging with FDG-PET compared to standard radiological methods for treatment response assessment during and after therapy in HL. While inducing HRS cells kill, chemotherapy also switches off the cytokine network, which activates the neoplastic HRS cells, thus turning off at the same time the functional activity of the ME cells. In conclusion, this double block of FDG uptake in these two different cellular compartments functions just as an “amplifier” of the PET signal, like an “HiFi” amplifier for the acoustic signal. The net result, in terms of imaging, is (1) an increase of detection power of FDG-PET and (2) an abrupt “on-off” switch of the PET images during and after therapy [11]. The scope of post-chemotherapy “Consolidation” radiotherapy (cRT) consists in eradicating the minority of the neoplastic tissue still metabolically active and surviving a very effective chemotherapy, very often inside a residual post-chemotherapy mass (RM). PET-guided cRT is aimed to cure persisting viable neoplastic tissue, while sparing undue toxicity to the largest fraction of PET-negative patients.

As a matter of fact, despite the evidence in the CT of a post-chemotherapy RM, as a possible harbinger of residual HL, the Negative Predictive Value (NPV) of a negative PET/CT proved very high, ranging between 80% and 100% [4,12], depending on the effectiveness of the chemotherapy regimen administered, thus ruling out the possibility of persisting disease in a PET-negative RM. The systematic use of PET/CT for end-of treatment (EoT) assessment of response in HL definitely made obsolete the concept of Response Complete Unconfirmed (RCU), proposed to define a complete response in which a CeCT-detected RM persisted [13], and introduced, at the same time, the concept of true metabolic response [14], which was finally incorporated in the Lugano criteria for lymphoma response assessment as “Complete Metabolic Response” [15].

The NPV of PET/CT after chemotherapy for HL proved strictly correlated with the effectiveness of the treatment delivered, ranging from 94% for BEACOPP escalated to 86% for VEBEP [16,17,18] (see below Section 2 and Section 3).

## 2. Residual Mass after ABVD Chemotherapy

In the pioneer experience of Naumann et al., 43 HL patients, presenting at baseline with classical bulky lesion (*n* = 30) or large nodal mass (*n* = 13), showed a residual mass (RM) after chemotherapy alone (*n =* 10) or chemoradiation (*n =* 33) [4]. The treatment response was assessed with FDG-PET: 39 had a negative and 4 a positive scan. The negative and positive predictive values on long-term treatment outcome were 100% and 25%, respectively. In 2015, Savage et al. [17], on behalf of the British Columbia Cancer Agency (BCCA), reported their retrospective experience in post-chemotherapy response assessment in a large cohort of HL patients in stage I bulky, or stage IIB–IVB, presenting at baseline with a large nodal mass, treated with six cycles of ABVD (also including patients in stage I bulky). All the patients included in this study had a negative end-of-treatment (EoT) PET, despite the presence of a residual mass (RM) detected on contrast-enhanced CT (CeCT) with the largest diameter measuring ≥ 2 cm [17]. Out of 316 patients included in the study, 264 (83.5%) in complete metabolic response had no further therapy, while 41 out of 52 (79%), showing a single or multiple FDG-avid persisting lesion, underwent consolidation radiotherapy. Unfortunately, no blinded, independent, central review for PET scan was planned, and the PET images were reported by the local PET sites according to traditional visual criteria, in which any residual FDG uptake was considered a harbinger of disease. With a median follow-up of 4.6 years (range 0.6–13.5 years), the 5-year freedom from treatment failure (FFTF) for the whole cohort was 83% and 5-years overall survival (OS) was 94.5%. Overall, patients with a negative EoT PET scan had a superior 5-years FFTF compared to those with a positive scan (89% vs. 56%, *p* < 0.00001). Remarkably, in the PET-negative group, there was no difference in outcome comparing the bulky (*n* = 112) and non-bulky (*n* = 152) subgroups, with a 5-years FFTF and OS of 89% vs. 88.5% (*p* = 0.50), and of 96% vs. 94% (*p* = 0.51), respectively. The authors concluded that: (1) ABVD-treated advanced-stage HL patients, including those with bulky disease with a negative PET scan after ABVD, have an excellent outcome without the need for additional consolidative RT; (2) thanks to a PET-guided approach, the Canadian Authors were able to significantly reduce the need for RT in the overall therapeutic strategy of HL.

**Figure 1:**
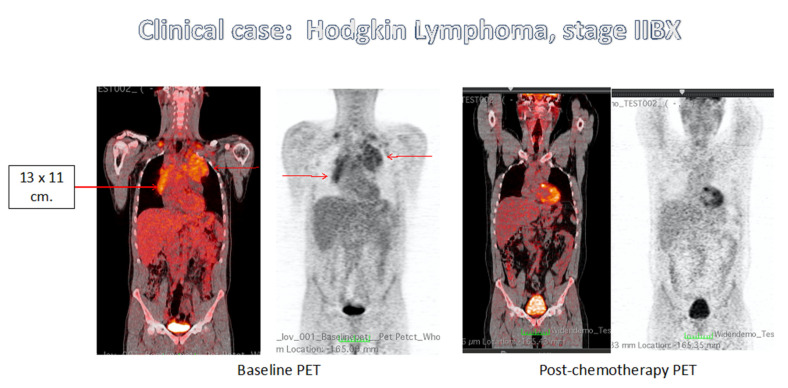
C.M., male, aged 38 years, admitted for evening fever >38 °C, night sweats, weight loss > 10 kg, cough, and enlarged cervical and supraclavicular nodes. On CT scan, a classic mediastinal bulky with a size of 13 × 11 cm was detected. Hemoglobin was 11.9 gr/dL, WBC 11,060 × 10^9^/L, Neutrophils 75%, Lymphocytes 15.6%, platelets 381 × 10^9^/L, LDH 287 U/L (NV: <460 UI/L). The Bone Marrow Biopsy was negative. The patient was in stage IIB X, with an IPS score of 2 (male, albumin < 4 gr/dL). The patient was enrolled in the HD 0607 clinical trial and treated with two ABVD cycles, with complete resolution of symptoms. A chest CT scan after two ABVD cycles showed a persisting mass of 6.2 × 4.4 cm and PET/CT was negative. The patient continued with ABVD for four further cycles, while still asymptomatic. A second chest CT scan after six ABVD cycles showed a persisting mass of 6 × 4.2 cm, and the EoT PET/CT was negative. The patient entered the randomized study of consolidation RT Vs. No Further Treatment (NFT), and he was randomized to NFT. Permission was obtained from Prof. Corrado Tarella on behalf of Italian lymphoma group GITIL (Gruppo Terapie Innovative nei Linfomi).

In 2008, the Italian HD 0607 trial was launched to assess the efficacy of an interim PET-adapted strategy in advanced stage Hodgkin lymphoma [19]. In this trial, patients with both a negative PET after two and six ABVD courses presenting at baseline with a Large Nodal Mass (LNM)—defined as a nodal mass with the largest diameter ≥ 5 cm measured in the CeCT scan performed for staging purposes, with or without evidence of a residual mass after chemotherapy (RM)—were randomly allocated to receive consolidation radiotherapy (cRT) or no further therapy (NFT). Both interim and EoT PET scans underwent blinded independent central review, and PET images were scored according to the 5-point Deauville scale [20]. Among 296 randomized patients, the largest diameter of LNM detected in the CeCT performed at baseline was 5–7 cm in 101 (34%)—Subgroup A; 8–10 cm in 96 (32%)—Subgroup B; while a classical bulky (diameter > 10 cm) was detected in 99 (33%)—Subgroup C. Two-hundred-and-eighty of them (88%) showed a post-chemotherapy RM. The median dose of consolidation radiotherapy delivered was 30.6 (range 24–36) Gy. After a median follow-up of 5.9 (0.5–10) years, the 6-years Progression-Free Survival (PFS) of patients randomized to cRT or NFT was: 91% (95% CI, 84% to 99%) and 95% (95% CI, 89% to 100%, *p* = 0.62) in Subgroup A; 98% (95% CI, 93% to 100%) and 90% (95% CI, 80% to 100%, *p* = 0.24) in Subgroup B; and 89% (95% CI, 81% to 98%) and 86% (95% CI, 77% to 96%, *p* = 0.53) in Subgroup C (classical bulky) [21] (Figure 1). The authors concluded that (1) consolidation radiotherapy does not improve treatment outcome, in terms of 6-years PFS and OS, in advanced-stage HL patients in complete metabolic response after ABVD, whatever the size of the LNM and whatever the Deauville score of the negative EoT PET (scored 1 to 3); (2) the pattern of recurrence in patients randomized to radiation and to NFT was similar, as in the radiation arm no imbalance of disease recurrence in new nodal areas compared to the areas involved at baseline was recorded; (3) the percentage of patients requiring cRT fell from 47% in patients with a PET-negative RM after ABVD to only 7%, in patients with a persisting positive EoT PET without evidence of disease progression or relapse. Very important, (4), the authors stressed that the most frequently detected LNM was in the mediastinum, which allowed to spare undue toxicity to the myocardium in up to 83% of the patients and to female breast tissue in nearly half of them, without compromising treatment outcome.

Quite recently, the results of the GHSG trial HD 17 were published [22]. In this trial, 1100 patients (aged 18–60 years) with newly diagnosed, histologically confirmed, stage IA–IIB classical, or nodular lymphocyte-predominant Hodgkin lymphoma, fulfilling the characteristics of early unfavorable or intermediate Hodgkin lymphoma according to the GHSG criteria, were enrolled at 224 hospitals and private practices in Germany, Switzerland, Austria, and the Netherlands. Eligible patients had to present at diagnosis with at least one of the following risk factors: a bulky mediastinal mass (i.e., measuring at least a third of the maximum transverse diameter of the thorax); extranodal involvement; either an erythrocyte sedimentation rate of 50 mm/h or higher without B-symptoms, or an erythrocyte sedimentation rate of 30 mm/h or higher with B-symptoms; or involvement of three or more nodal areas. Overall, 1100 patients were enrolled, and 979 patients were randomly allocated to the standard combined-modality treatment group (*n =* 486) consisting of the 2 + 2 regimen (two cycles of escalated BEACOPP plus two cycles of ABVD), followed by 30 Gy involved-node radiotherapy (INRT), whatever the results of post-chemotherapy PET/CT or in the EoT PET-guided therapy (*n* = 493), consisting of the 2 + 2 regimen for all the patients randomized in this arm, followed by 30 Gy INRT only to patients with a positive post-chemotherapy PET. At a median follow-up of 46.2 months (IQR 32.7–61.2), the 5-year PFS was 97.3% (95% CI 94.5–98.7) in the standard combined-modality treatment group and 95.1% (92.0–97.0) in the PET4-guided treatment group (hazard ratio 0.523 (95% CI 0.226–1.211)). The between-group difference was 2.2% (95% CI −0.9 to 5.3) and fulfilled the criteria for a non-inferiority result. A descriptive post-hoc subgroup analysis of patients in the per-protocol analysis population with initial bulky disease or a large mediastinal tumor showed non-inferiority of PET-guided treatment in this subgroup of patients, with a 5-year progression-free survival of 96% (95% CI 91.0–98.2) in the combined-modality treatment group (*n* = 225), and 96.5% (93.1 to 98.2) in the PET4-guided treatment group (*n* = 267; difference 0.5% (95% CI –3.6 to 4.6)). A post-chemotherapy RM > 2.5 cm was found in 32% (standard arm) and 35% (experimental arm) of the patients. Post-hoc sensitivity analyses in PET4-negative patients supported this finding; 5-year progression-free survival in the combined-modality treatment group (*n* = 122) was 96.7% (87.3 to 99.2) and 97.0% (92.2 to 98.9) in the PET4-guided treatment group (*n* = 157), with a difference of 0.4% (–5.0 to 5.8) between the two groups.

## 3. Residual Mass after BEACOPP_escalated_ Chemotherapy in Advanced Hodgkin lymphoma

The dilemma of the interpretation of a RM after BEACOPP_escalated_ chemotherapy evolved similarly, albeit slower than after ABVD. In the pre-PET/CT era, the German Hodgkin Study Group (GHSG) considered any RM [23] or, later, a RM measuring ≥ 1.5 cm [24], as an harbinger of residual disease and, therefore, subjected to consolidative radiotherapy at a dose 30–40 Gy. About 60–65% of patients responding to BEACOPP_escalated_ therapy had a RM ≥ 1.5 at the end of treatment, mainly in the mediastinum, but despite consolidation radiotherapy, few of these patients eventually relapsed [24,25].

In the randomized HD 12 trial conducted on 730 advanced-stage HL patients, the first attempts to omit radiotherapy in patients with a CeCT-detected post-chemotherapy RM ≥ 1.5 cm, with an otherwise incomplete or partial response (CR or PR) after 8 BEACOPP_escalated_ or 4 BEACOPP_escalated_ + 4 BEACOPP_baseline_ were unsuccessful, as an inferior Freedom from Treatment Failure (FFTF) was recorded in patients treated with chemotherapy alone compared to those undergoing combined modality treatment (difference: 5.8%; 95% CI: 10.7% to 1.0%) [24]. The authors concluded that the trial showed clear limitations of CeCT as a tool to select patients needing consolidative RT.

The first results on the role of FDG-PET in RM interpretation after BEACOPP, reported by Weihrauch et al. in 2001 in 28 patients with advanced HL, showed only a moderate positive predictive value (PPV) of 60% and a much higher negative predictive value (NPV) of 95%, as a likely consequence of the conservative definition of a negative PET result (any visual focally increased FDG uptake was considered positive) [25]. The introduction of the criteria of the Imaging Subcommittee of the International Harmonization Project in Lymphoma (IHPC) in 2007 and, later, of the 5-point Deauville scale (DS) [26], dramatically improved the interpretation of the PET/CT results and pushed forward the understanding of the meaning of a RM after BEACOPP_escalated_ chemotherapy.

In the GHSG HD15 trial that enrolled 2182 HL patients, only patients with a post-chemotherapy RM measuring ≥ 2.5 cm in the CeCT and showing a focal FDG uptake in EoT PET received additional radiotherapy with 30 Gy, while patients with a negative PET or those without a RM did not [16]. The criterion for a negative EoT PET was, according to IHPC criteria, the presence of a residual FDG uptake with an intensity not superior to that of the surrounding background or, in the case of a RM with a larger diameter ≥ 2 cm, not higher than that of the FDG uptake measured in the mediastinal blood pool [27]. Out of 739 patients with a RM ≥ 2.5, the PET was positive in 191 (26%). The NPV was very high: 94.1% at 12 months (95% CI 92.1–96.1), thus allowing a marked reduction of the percentage of patients requiring consolidation RT from 71% recorded in the HD 9 trial to only 11% [16,23]. In other words, patients in partial remission by CeCT with a PET-negative RM after chemotherapy had a prognosis very similar to the patients without RM (4-year progression-free survival of 92.1%). FDG PET/CT assessment also showed that prognosis of patients with a positive EoT PET before consolidative RT was worse than that recorded in patients with PET-negative RM treated with chemotherapy alone without RT (PFS at 48 months of 86.2% compared to 92.6%; 95% C.I. for difference 0.9–12.0, log rank *p* = 0.022).

The definition of negative EoT PET used in the HD15 trial corresponded to DS score of 1–2 (DS1,2). In ABVD-treated patients, not only DS1–2 but also DS3 at the end of chemotherapy was consistent with CMR. However, there was no formal proof that this held true also in patients treated with BEACOPP_escalated,_ until the results of EoT PET reappraisal in the more recent trial of GHSG (HD18) in patients with advanced HL were available. In this trial, the same conservative definition for a positive/negative interim and end-of-therapy PET as in HD15 trial was originally used, adopting a Deauville score ≥ 3 (DS3) as a result for a positive scan. Accordingly, a PET positive RM measuring ≥ 2.5 cm in the EoT CeCT was treated with consolidating radiotherapy with a dose of 30 Gy [28]. However, in a subsequent analysis of interim PET after 2 BEACOPP_escalated_ in which a Deauville score ≥ 4 for a positive PET was adopted, showed that PFS at 3 years (95.9%) was not different for patients with DS1–2 (92.2%) [29]. Unfortunately, the same analysis was not performed for EOT PET, due to a low number of cases. Nevertheless, the ≥4 cutoff for a positive EoT PET was adopted during the course of the HD18 trial in the last few years to guide consolidation RT, and the same cutoff was adopted in the current recommendations of GHSG [30] to restrict the use of consolidation radiotherapy on a RM after BEACOPP _escalated_, regardless of the size of the residual mass.

## 4. Interpretation Criteria for PET/CT at the End of Therapy in Patients with a CT-Detected Residual Mass

In 2014, the so-called Lugano classification indicated the criteria for response assessment in Hodgkin and non-Hodgkin lymphoma patients undergoing restaging with FDG-PET [15]. Visual response assessment according to the Deauville 5-point scale is preferred to semiquantitative metrics with Standardized Uptake Value (SUV) [31]. Complete metabolic response is defined by a PET scan scored 1 to 3, while a score of 4 to 5 identifies residual disease. However, in the presence of a RM these criteria proved imperfect. In a recent retrospective study in 169 patients with advanced-stage HL and a RM with a size ranging between 1 and 4.2 cm, a DS score of 1 was found in 99 patients, a score of 2 in 3, a score of 3 in 24, a score of 4 in 32, and a score of 5 in 11 patients. The 43 patients with positive PET at restaging received imaging-guided biopsy; histology showed malignancy (HL) in 100% of DS 5 scores and only in 12.5% of DS 4 scores [32].

Nonetheless, DS continues to be the preferred interpretation tool in EoT FDG-PET/CT in Hodgkin lymphoma, while the semiquantitative approach by ΔSUVmax has been proposed with conflicting results in adult [33] and pediatric [34] HL patients.

## 5. Texture Analysis—Radiomics in Hodgkin lymphoma

Cancer cells show consistent heterogeneity with variable cell dimension and morphology, phenotype profiles, gene expression, metabolism, motility, proliferation, and metastatic potential; this complex phenomenon also reflects genome instability and epigenetic variation, and it has been associated with differences in outcome across several cancer types. Primary Mediastinal B-Cell Lymphoma (PMBCL) very often presents with a very large single mass in the upper anterior mediastinum; it putatively derives from a thymic B cell-precursor, and it could be considered to lie on a pathobiologic spectrum of diseases, the so-called Mediastinal Grey Zone Lymphomas, including Nodular sclerosis Hodgkin lymphoma (typically CD15- and CD30-positive) and PMBCL itself (CD20-positive) [35]. High heterogeneity of intra-tumoral Fluoro-deoxy-glucose (FDG) uptake distribution on PET/CT has been suggested as a possible marker of chemoresistance in solid tumors. In 2018, Ceriani et al. investigated the prognostic value of metabolic heterogeneity (MH) in 103 PMBCL patients prospectively enrolled in the International Extranodal Lymphoma Study Group (IELSG) 26 study, aimed at clarifying the role of PET in this lymphoma subtype [36]. MH was estimated using the area under curve of cumulative standardized uptake value-volume histogram (AUC-CSH) method. Progression-free survival at 5 years was 94% vs. 73% in low and high-MH groups, respectively (*p* < 0.0001).

In the early 60s of the last century, the pioneer studies on the role of laparotomy in the surgical staging of HL demonstrated the tumor spread had a crucial prognostic role in this disease [37]. More than 50 years later, during the 2021 International Congress on Malignant Lymphoma in Lugano, Durmo et al. presented their retrospective experience on the prognostic role of lesion dissemination (DMAX) features in a retrospective cohort of advanced-stage HL treated in a single Institution [38]. DMAX was defined as the largest distance between two lesions measured from FDG-PET/CT scans.

Briefly, all lesions detected in baseline PET/CT were semiautomatically segmented, and the centroid of each lesion was automatically obtained and considered as the lesion location. The distances between all pairs of lesions were calculated and DMAX was obtained for each patient. DMAX was then dichotomized according to the median value within our cohort. In univariate analysis, International Prognostic Score (IPS) > 2 (HR 2.20 CI 1.26–3.87, *p* = 0.006), a positive interim PET (HR 3.32 CI 1.73–6.39, *p* < 0.001), and DMAX > 20 cm (HR 2.42 CI 1.31–4.47, *p* = 0.005) were all associated with shorter PFS.

The rapidly increasing amount of information generated by medical imaging is currently assessed for its prognostic and predictive role in treatment outcome in the so-called machine learning process. In this process, mathematical algorithms extract single or multiple morphologic parameters with a still unknown prognostic role from large data sets generated from prospective clinical trials. New predictive or prognostic models are thus generated moving from these “new” biomarkers retrospectively extracted from large databases to be validated prospectively in well-designed clinical trials [39].

## 6. Residual Mass after Immune Checkpoint Inhibitors Therapy

The interpretation of a RM during or after therapy with immune check point inhibitors (CPI) is even more challenging for at least two reasons. First, because the length of treatment with CPI is not defined and most patients are treated until progression, thus being assessed for the treatment response during, and not after, CPI administration. Second, more importantly, because of the mechanism of action of these drugs, consisting in reactivation of endogenous tumoricidal immune activity that may lead to transient or persisting pseudo-progressions or partial responses that remain partial overtime. As a matter of fact, tumor imaging with FDG-PET during CPI administration yielded the following changes: (1) an increase in the size of overall tumor burden in the first 6 months of therapy, (2) an isolated increase in the FDG uptake without a change in lesion size or (3) even the appearance of new lesions [40]. Therefore, a provisional modification of the Lugano criteria was proposed through the introduction of a new response category termed indeterminate response (IR) to avoid premature cessation of effective treatment with CPI in patients that might be classified otherwise as having progressive disease [41]. This proposed classification, which at a first glance sounds like an oxymoron (how to determine a treatment response with indeterminate criteria) requires after the first assessment, to repeat a second assessment within 12 weeks from the first one, and reevaluation of the original findings to determine whether they portrayed a true or a pseudo progression. However, these criteria have never been prospectively evaluated and are not reported in real life setting [42] or clinical trials [43,44,45]. Nevertheless, the dilemma on how to assess the response of HL patients during immunotherapy was faced by investigators of the Checkmate-205 trial in which de-novo advanced stage HL patients treated with nivolumab in combination with AVD, despite a positive EoT PET, achieved durable remissions [46]. Recently, a retrospective study of 45 patients from 34 institutions was conducted to assess the role of CeCT and PET/CT in response assessment to Nivolumab with relation to overall survival [47]. Patients were examined with CT and FDG PET/CT at two time points: after a median of 2 months (primary) and 6.7 months (secondary assessment) after the start of Nivolumab treatment. CeCT and PET/CT were assessed according to the 2014 Lugano Classification [15] and the 2016 revised LYRIC criteria [41]. The authors found that 2-year OS in patients with CMR at primary assessment was 100%, including those failing to achieve CR on CeCT performed at the same timepoint. Additionally, all patients classified as having progressive disease at primary assessment using CeCT (12 of 45 patients) were classified as having progressive metabolic disease at primary assessment using PET/CT. In fact, PET/CT reclassified as CMR 11/45 in the first and 11/36 in the second turnaround of CT assessment. The concordance between CeCT and PET/CT second assessment was better compared to first assessment (kappa Cohen 0.78 compared to 0.62). The fact that PET/CT outperformed CeCT, identifying a clinically relevant response earlier, is consistent with all previous experience with PET/CT in HL. However, these preliminary data should be taken with caution due to the concerns on the confounding message of two different and opposite physio pathological mechanisms both perceived with a positive signal on PET: The first due to a restored immune cell-mediated tumor lysis, the second to a persisting viable neoplastic tissue [40]. Indeed, some of the patients in preliminary reports [40] showed unique behavior or signs of pseudo-progression. The rate of patients with pseudo progressive lesions was 16.7% of early progressive patients on primary assessment using CT (2/12), 12.5% of early progressive patients on primary assessment using PET/CT (2/16), and 4.4% of the overall cohort (2/45). However, despite a lack of overt progression on CT (12) or on PET/CT (16) on the first assessment, subsequently a diagnosis of true progressive disease became undeniable, meaning the abnormal findings detected on CT and on PET, which were classified in the former scans as IR according to LYRIC criteria, were indeed true progressions. Moreover, the authors observed that few cases with pseudoprogressive lesions involved only one anatomic site (e.g., retroperitoneal lymph nodes, liver), but otherwise most of these patients met unequivocal criteria of disease progression [47]. Altogether, the above reported findings in HL patients assessed during immunotherapy suggest that a substantial number of IRs per LYRIC are indeed true HL progression.

Nevertheless, false positive results of PET/CT remain a challenge that partially may explain the equivalent outcome for patients who underwent ASCT directly after anti-PD-1 therapy regardless of pre-transplant PET result with 18-month PFS of 85% in PET-positive and PET-negative patients [48]. These patients may require histopathology verification as it was done in four patients treated with Brentuximab Vedotin and Nivolumab in whom despite PET-positive lesions (scored as DS 4 or 5), no evidence of lymphoma was found on biopsy [49]. A promising possible solution is the association of PET imaging with biomarkers such as the circulating cell-free tumor DNA (cfDNA), which proved able to “mirror” the total tumor burden with a very high specificity. A linear relationship with metabolic tumor volume (MTV) measured with PET/CT and cfDNA value in the plasma was recently demonstrated in longitudinal studies of HL patients at diagnosis, interim during treatment, after treatment end, and at disease relapse [50]. CfDNA assay in the presence of temporary “flares” of PET/CT could be the ideal biomarker to improve the overall accuracy for response assessment during and after CPI administration.

## 7. PET/MRI for Lymphoma Staging and Restaging

To date, no major advantages have been shown for lymphoma staging and restaging with PET coupled with Magnetic Resonance Imaging (PET/MRI) compared to PET/CT. In a recently published study, 66 consecutive patients with histologically proven Hodgkin or non-Hodgkin lymphoma (NHL) were prospectively evaluated [51]. Each patient had whole body PET/CT, followed by whole body PET/MR. Ninety-five nodal and eight extranodal sites were identified on both PET/CT and PET/ MRI. In addition, three nodal and one extranodal sites were identified on PET/MRI. For positive lesion detection, reader agreement in PET/MR was perfect between the two readers and almost perfect between PET/CT and PET/MRI (k > 0.978). Intermodality agreement between PET/CT and PET/MRI was also near-perfect to perfect for staging/disease status (0.979–1.000), but there were no advantages of PET/MRI over PET/CT. In another study on 18 patients including 5 HL and 13 NHL, the SUVmax from FDG-PET/MR (21.3 ± 2.07) vs. FDG-PET/CT (mean 23.2 ± 2.8) demonstrated a strongly positive correlation (rs = 0.95 (0.94, 0.99); *p* < 0.0001) [52]. There was no correlation found between Apparent Diffusion Coefficient (ADC) and SUVmax from FDG-PET/MR (r = 0.17 (−0.07, 0.66); *p* = 0.09).

The calculation of ADC of free water molecules movement across the extra cellular space has been advocated as an indirect parameter to measure the tissue cellular density and, consequently, to assess tumor response with MRI in oncology [53]. In fact, dynamic longitudinal studies in animal models have shown early ADC changes after chemotherapy onset as early as in day 1 [53], and more consistently in the days 4.5–7 after chemotherapy administration [54].

However, the main problems are reproducibility and reliability of ADC: ADC values tend to vary when different scan parameters or methods of measurement are applied, limiting reproducibility and comparability between centers. Intra-patient reproducibility of ADC proved indeed sub-optimal, with a range of relative difference of 4–9%, and the difference exceeded 3–5% while repeated measures of the same subjects using the same imager were performed; therefore, substantial errors in the absolute ADC values appear to be unavoidable [55]. At this writing, there are no data of direct comparison of PET/CT and PET/MRI in lymphoma restaging.

**Figure 2:**
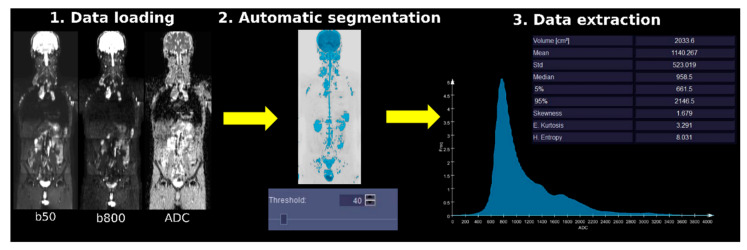
Whole-body diffusion weighted imaging (WB-DWI) image analysis and data extraction process using syngo, via Frontier MR Total Tumor Load software. Reprinted from [56].

Quite recently, a new MRI-derived parameter has been proposed, the Total Tumor Load (TTL) for treatment response assessment at the end of therapy. This marker originates from the promising results obtained by WB-DWI and ADC in multifocal disease, such as bone metastases and multiple myeloma, and the resulting need to dispose of an efficient tool able to evaluate this kind of lesion. This tool harnesses a threshold-based segmentation algorithm on MRI whole-body diffusion-weighted images, in order to identify regions of disease, while providing both the overall diffusion tumor volume and the histogram metrics of the corresponding computed ADC maps [56]. A high b-value image (acquired or computed) is used as input, in order to maximize the contrast between lesions and healthy tissue. This method was assessed in a cohort of 20 HL patients who underwent PET/CT followed by PET/MRI with WB-DWI at baseline before any treatment (T0), after two chemotherapy cycles (T1), and at the end-of-treatment (T2). The analysis of WB-DWI images at each time point was performed using the syngo.via Frontier MR Total Tumor Load released research prototype v1.3.3 (Siemens Healthineers, Erlangen, Germany). The MRI Total Tumor Load was calculated at six segmentation thresholds (5%, 10%, 20%, 40%, 60%, and 80%) and using Lugano criteria applied on PET/CT images as reference standard (see Figure 2).

Using the WB-DWI images as input (1. Data Loading step), b800 images were automatically segmented, setting a signal intensity threshold (e.g., 40%) at each WB-DWI acquisition time point (e.g., T0). No subsequent mask editing was made (2. Automatic segmentation). The overall mask volume (diffusion volume, DV) and the corresponding apparent diffusion coefficient (ADC) histogram metrics associated with the masked volume were extracted (3. Data extraction step). Yellow arrows link the three processing steps.

Concerning prediction of EOT response, the Diffusion Volume (DV) at 40% and 60% thresholds was found to be significantly higher in PMR patients than in CMR patients, with an AUC of 96%, and a sensitivity, specificity, and overall accuracy of 100%, 93%, and 96%, respectively. The obtained results supported the hypothesis that Diffusion Volume and its related histogram-based Apparent Diffusion Coefficient statistics could be useful in prediction and assessments of HL response to therapy. However, this method is still far away from being adopted in clinical practice, due to a lack of standardization.

## 8. Discussion

**Figure 3:**
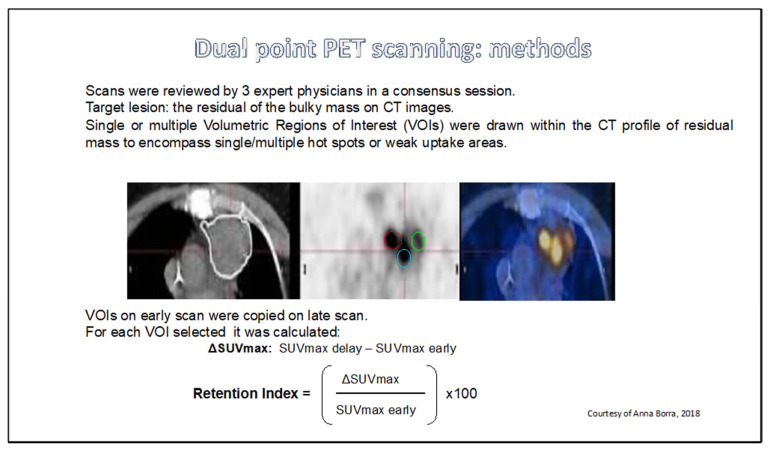
Dual-point PET scanning: methodology. Adapted with permission from Borra et al. [63] by doctor Borra’s permission. Copyright 2018 e-Century Publishing Corporation.

Despite the determinant progress in evaluating response to treatment and interpretation of post-chemotherapy RM in HL patients with functional imaging with PET, this approach has some limitations, mainly due to false positive results, which are more evident especially, but not exclusively, during CPI treatment. One of the well-known pitfalls is the post-chemotherapy thymus hyperplasia, which occurs in about 5% of patients [57]. In most cases, the FDG uptake is moderate (SUVmax < 4), and the morphology of residual uptake is suggestive of the thymus anatomic structure, but in some cases the intensity of FDG uptake might be higher, probably because of the inefficient immunity of the host against the tumor [58]. A similar mechanism seems to underpin the persistent hilar and mediastinal lymph nodes FDG uptake not related to HL. Examples include sarcoidosis that is often associated with lymphomas and more importantly granulomatosis that can be induced by treatment especially with immune check point inhibitors [59,60]. Therefore, persistence of PET-positive residual lymph nodes, especially in mediastinum, is not sufficient to confirm resistant disease especially in symmetric mediastinal-hilar lymph node uptake. These patients need, rather, a follow-up or biopsy especially in the case of an atypical uptake. As a matter of fact, only in about half [61] or even less frequently [28], of the FDG-avid lymph nodes persisting in the mediastinum after treatment for HL are due to a histologically-proven persisting HL. The issue of selectively imaging the neoplastic tissue while “subtracting” the inflammatory tissue from the background has been explored in the past by the so-called “dual-point PET imaging”, consisting of two different scans performed 60 and 120 min after the same FDG dose injection in a patient showing a post-chemotherapy RM. Briefly, this technique was underpinned by the observation that neoplastic tissue has a much higher FDG “trapping” due to the lack of glucose (and FDG-glucose)-6-phosphate phosphatase in neoplastic cells, leading to a FDG accumulation inside the cytoplasm of these cells increasing overtime, in contrast with the inflammatory tissue, in which a certain degree of FDG spontaneous elution from the cell is observed, also increasing after FDG injection as the time goes by [62]. This technique has allowed to reclassify some persisting FDG uptake in HL post-chemotherapy RM as “non neoplastic” but not in all the patients [63] (Figure 3).

Finally, in the future, a combination of imaging studies with new imaging techniques such as radiomics [36], or with a biomarker such as tumor-associated cell-free DNA, could further decrease or completely abrogate the margin of underdetermined interpretation of residual mass after chemotherapy in Hodgkin lymphoma [64].

## 9. Conclusions

End-of therapy PET scan is now considered the optimal tool for HL restaging after first-line chemotherapy both in early and advanced stage disease, with or without evidence of a residual mass (RM), with a very high negative predictive value and a variable positive predictive value. In the few cases (5–10%) with a PET-positive RM, end-of therapy PET proved the best tool to guide consolidation radiotherapy, thus allowing to spare toxicity in a large number of patients. In this clinical context, PET/MRI or Diffusion Weighted Nuclear Magnetic Resonance did not show any advantage over PET/CT. On the contrary, End-of-Therapy PET scan proved to be a sub-optimal tool to assess treatment response with immune checkpoint inhibitors. In future, the association of PET/CT with specific biomarkers of tumor burden such as cell-free tumor DNA assay could increase the overall accuracy of the response evaluation on this new class of drugs.

## Data Availability

No new data were created or analyzed in this study. Data sharing is not applicable to this article.
